# Retraction Note: Ferroportin in the progression and prognosis of hepatocellular carcinoma

**DOI:** 10.1186/s40001-015-0132-6

**Published:** 2015-03-26

**Authors:** Qin Wang, Jun Zhou, Dewu Zhong, Qunwei Wang, Jiangsheng Huang

**Affiliations:** Department of Gynecology, The Second Xiangya Hospital of Central South University, Changsha, 410011 Hunan Province China; Department of Minimal Invasive surgery, The Second Xiangya Hospital of Central South University, 139 Renmin Middle Road, Changsha, 410011 Hunan Province China; Department of General Surgery, The Second Xiangya Hospital of Central South UniversityChangsha, Changsha, 410011 Hunan Province China

## Retraction

The Publisher and Editor regretfully retract this article [1] because the peer-review process was inappropriately influenced and compromised. As a result, the scientific integrity of the article cannot be guaranteed. A systematic and detailed investigation suggests that a third party was involved in supplying fabricated details of potential peer reviewers for a large number of manuscripts submitted to different journals. In accordance with recommendations from COPE we have retracted all affected published articles, including this one. It was not possible to determine beyond doubt that the authors of this particular article were aware of any third party attempts to manipulate peer review of their manuscript.
